# Supplementary material for: HIVprotI: an integrated web based platform for prediction and design of HIV proteins inhibitors
Source: J Cheminform. 2018 Mar 9;10:12. doi: 10.1186/s13321-018-0266-y (PMC5845081; doi:10.1186/s13321-018-0266-y)
Supplement: Supplementary file 2 — Additional file 2. Source code of HIVProtI web server. [file 13321_2018_266_MOESM2_ESM.zip › HIVprotI_Source-code/hivprotI/weka-3-6-6/doc/weka/gui/arffviewer/ArffViewerMainPanel.html]

ArffViewerMainPanel


---


|  |  |  |
| --- | --- | --- |
| **Overview** \| **Package** \| **Class** \| **Tree** \| **Deprecated** \| **Index** \| **Help** \| **Weka's home** | |  |
| **PREV CLASS**   **NEXT CLASS** | **FRAMES**    **NO FRAMES**     **All Classes** |  |
| SUMMARY: NESTED \| FIELD \| CONSTR \| METHOD | DETAIL: FIELD \| CONSTR \| METHOD |  |


---


## weka.gui.arffviewer Class ArffViewerMainPanel

```
java.lang.Object
  java.awt.Component
      java.awt.Container
          javax.swing.JComponent
              javax.swing.JPanel
                  weka.gui.arffviewer.ArffViewerMainPanel
```

**All Implemented Interfaces:**: java.awt.event.ActionListener, java.awt.image.ImageObserver, java.awt.MenuContainer, java.io.Serializable, java.util.EventListener, javax.accessibility.Accessible, javax.swing.event.ChangeListener

---

``` public class ArffViewerMainPanel extends javax.swing.JPanel implements java.awt.event.ActionListener, javax.swing.event.ChangeListener ```

The main panel of the ArffViewer. It has a reference to the menu, that an
implementing JFrame only needs to add via the setJMenuBar(JMenuBar) method.

**Version:**
:   $Revision: 7059 $

**Author:**
:   FracPete (fracpete at waikato dot ac dot nz)

**See Also:**: Serialized Form

---

**Nested Class Summary**

**Nested classes/interfaces inherited from class javax.swing.JComponent**

`javax.swing.JComponent.AccessibleJComponent`

**Nested classes/interfaces inherited from class java.awt.Component**

`java.awt.Component.BaselineResizeBehavior`


| **Field Summary** | |
| --- | --- |
| `static int` | `DEFAULT_HEIGHT`             the default for height |
| `static int` | `DEFAULT_LEFT`             the default for left |
| `static int` | `DEFAULT_TOP`             the default for top |
| `static int` | `DEFAULT_WIDTH`             the default for width |
| `static int` | `HEIGHT`             default height |
| `static int` | `WIDTH`             default width |

**Fields inherited from class javax.swing.JComponent**

`TOOL_TIP_TEXT_KEY, UNDEFINED_CONDITION, WHEN_ANCESTOR_OF_FOCUSED_COMPONENT, WHEN_FOCUSED, WHEN_IN_FOCUSED_WINDOW`

**Fields inherited from class java.awt.Component**

`BOTTOM_ALIGNMENT, CENTER_ALIGNMENT, LEFT_ALIGNMENT, RIGHT_ALIGNMENT, TOP_ALIGNMENT`

**Fields inherited from interface java.awt.image.ImageObserver**

`ABORT, ALLBITS, ERROR, FRAMEBITS, PROPERTIES, SOMEBITS`


**Constructor Summary**

`ArffViewerMainPanel(java.awt.Container parentFrame)`
  
          initializes the object


| **Method Summary** | |
| --- | --- |
| `void` | `actionPerformed(java.awt.event.ActionEvent e)`             invoked when an action occurs |
| `void` | `attributeAsClass()`             sets the current selected Attribute as class attribute, i.e. |
| `void` | `clearSearch()`             clears the search, i.e. |
| `void` | `close()`             closes the window, i.e., if the parent is not null and implements the WindowListener interface it calls the windowClosing method |
| `void` | `closeAllFiles()`             closes all open files |
| `void` | `closeFile()`             closes the current tab |
| `void` | `closeFile(boolean showCancel)`             closes the current tab |
| `void` | `copyContent()`             copies the content of the selection to the clipboard |
| `void` | `deleteAttribute(boolean multiple)`             deletes the current selected Attribute or several chosen ones |
| `void` | `deleteInstance(boolean multiple)`             deletes the current selected Instance or several chosen ones |
| `boolean` | `getConfirmExit()`             returns the setting of whether to display a confirm messagebox or not on exit |
| `java.lang.String` | `getCurrentFilename()`             returns the filename of the current tab |
| `int` | `getCurrentIndex()`             returns the currently selected tab index |
| `ArffPanel` | `getCurrentPanel()`             returns the currently selected panel |
| `boolean` | `getExitOnClose()`             returns TRUE if a System.exit(0) is done on a close |
| `java.lang.String` | `getFilename(int index)`             returns the filename of the specified panel |
| `java.lang.String` | `getFrameTitle()`             returns the title (incl. |
| `javax.swing.JMenuBar` | `getMenu()`             returns the menu bar to be added in a frame |
| `ArffPanel` | `getPanel(int index)`             returns the specified panel, `null` if index is out of bounds |
| `int` | `getPanelCount()`             returns the number of panels currently open |
| `javax.swing.JFrame` | `getParentFrame()`             returns the parent frame, if it's a JFrame, otherwise null |
| `javax.swing.JInternalFrame` | `getParentInternalFrame()`             returns the parent frame, if it's a JInternalFrame, otherwise null |
| `javax.swing.JTabbedPane` | `getTabbedPane()`             returns the tabbedpane instance |
| `boolean` | `isPanelSelected()`             checks whether a panel is currently selected |
| `void` | `loadFile()`             loads the specified file into the table |
| `void` | `loadFile(java.lang.String filename)`             loads the specified file |
| `void` | `refresh()`             validates and repaints the frame |
| `void` | `renameAttribute()`             renames the current selected Attribute |
| `void` | `saveFile()`             saves the current data into a file |
| `void` | `saveFileAs()`             saves the current data into a new file |
| `void` | `search()`             searches for a string in the cells |
| `void` | `setConfirmExit(boolean confirm)`             whether to present a MessageBox on Exit or not |
| `void` | `setCurrentFilename(java.lang.String filename)`             sets the filename of the current tab |
| `void` | `setExitOnClose(boolean value)`             whether to do a System.exit(0) on close |
| `void` | `setFilename(int index, java.lang.String filename)`             sets the filename of the specified panel |
| `void` | `setOptimalColWidths()`             sets the optimal column width for all columns |
| `void` | `setParent(java.awt.Container value)`             sets the new parent frame |
| `java.lang.String` | `showAttributes()`             displays all the attributes, returns the selected item or NULL if canceled |
| `void` | `showProperties()`             displays some properties of the instances |
| `void` | `showValues()`             displays all the distinct values for an attribute |
| `void` | `sortInstances()`             sorts the current selected attribute |
| `void` | `stateChanged(javax.swing.event.ChangeEvent e)`             Invoked when the target of the listener has changed its state. |
| `java.lang.String` | `toString()`             returns only the classname |
| `void` | `undo()`             undoes the last action |
| `void` | `updateFrameTitle()`             sets the title of the parent frame, if one was provided |

**Methods inherited from class javax.swing.JPanel**

`getAccessibleContext, getUI, getUIClassID, setUI, updateUI`

**Methods inherited from class javax.swing.JComponent**

`addAncestorListener, addNotify, addVetoableChangeListener, computeVisibleRect, contains, createToolTip, disable, enable, firePropertyChange, firePropertyChange, firePropertyChange, getActionForKeyStroke, getActionMap, getAlignmentX, getAlignmentY, getAncestorListeners, getAutoscrolls, getBaseline, getBaselineResizeBehavior, getBorder, getBounds, getClientProperty, getComponentPopupMenu, getConditionForKeyStroke, getDebugGraphicsOptions, getDefaultLocale, getFontMetrics, getGraphics, getHeight, getInheritsPopupMenu, getInputMap, getInputMap, getInputVerifier, getInsets, getInsets, getListeners, getLocation, getMaximumSize, getMinimumSize, getNextFocusableComponent, getPopupLocation, getPreferredSize, getRegisteredKeyStrokes, getRootPane, getSize, getToolTipLocation, getToolTipText, getToolTipText, getTopLevelAncestor, getTransferHandler, getVerifyInputWhenFocusTarget, getVetoableChangeListeners, getVisibleRect, getWidth, getX, getY, grabFocus, isDoubleBuffered, isLightweightComponent, isManagingFocus, isOpaque, isOptimizedDrawingEnabled, isPaintingForPrint, isPaintingTile, isRequestFocusEnabled, isValidateRoot, paint, paintImmediately, paintImmediately, print, printAll, putClientProperty, registerKeyboardAction, registerKeyboardAction, removeAncestorListener, removeNotify, removeVetoableChangeListener, repaint, repaint, requestDefaultFocus, requestFocus, requestFocus, requestFocusInWindow, resetKeyboardActions, reshape, revalidate, scrollRectToVisible, setActionMap, setAlignmentX, setAlignmentY, setAutoscrolls, setBackground, setBorder, setComponentPopupMenu, setDebugGraphicsOptions, setDefaultLocale, setDoubleBuffered, setEnabled, setFocusTraversalKeys, setFont, setForeground, setInheritsPopupMenu, setInputMap, setInputVerifier, setMaximumSize, setMinimumSize, setNextFocusableComponent, setOpaque, setPreferredSize, setRequestFocusEnabled, setToolTipText, setTransferHandler, setVerifyInputWhenFocusTarget, setVisible, unregisterKeyboardAction, update`

**Methods inherited from class java.awt.Container**

`add, add, add, add, add, addContainerListener, addPropertyChangeListener, addPropertyChangeListener, applyComponentOrientation, areFocusTraversalKeysSet, countComponents, deliverEvent, doLayout, findComponentAt, findComponentAt, getComponent, getComponentAt, getComponentAt, getComponentCount, getComponents, getComponentZOrder, getContainerListeners, getFocusTraversalKeys, getFocusTraversalPolicy, getLayout, getMousePosition, insets, invalidate, isAncestorOf, isFocusCycleRoot, isFocusCycleRoot, isFocusTraversalPolicyProvider, isFocusTraversalPolicySet, layout, list, list, locate, minimumSize, paintComponents, preferredSize, printComponents, remove, remove, removeAll, removeContainerListener, setComponentZOrder, setFocusCycleRoot, setFocusTraversalPolicy, setFocusTraversalPolicyProvider, setLayout, transferFocusBackward, transferFocusDownCycle, validate`

**Methods inherited from class java.awt.Component**

`action, add, addComponentListener, addFocusListener, addHierarchyBoundsListener, addHierarchyListener, addInputMethodListener, addKeyListener, addMouseListener, addMouseMotionListener, addMouseWheelListener, bounds, checkImage, checkImage, contains, createImage, createImage, createVolatileImage, createVolatileImage, dispatchEvent, enable, enableInputMethods, firePropertyChange, firePropertyChange, firePropertyChange, firePropertyChange, firePropertyChange, getBackground, getBounds, getColorModel, getComponentListeners, getComponentOrientation, getCursor, getDropTarget, getFocusCycleRootAncestor, getFocusListeners, getFocusTraversalKeysEnabled, getFont, getForeground, getGraphicsConfiguration, getHierarchyBoundsListeners, getHierarchyListeners, getIgnoreRepaint, getInputContext, getInputMethodListeners, getInputMethodRequests, getKeyListeners, getLocale, getLocation, getLocationOnScreen, getMouseListeners, getMouseMotionListeners, getMousePosition, getMouseWheelListeners, getName, getParent, getPeer, getPropertyChangeListeners, getPropertyChangeListeners, getSize, getToolkit, getTreeLock, gotFocus, handleEvent, hasFocus, hide, imageUpdate, inside, isBackgroundSet, isCursorSet, isDisplayable, isEnabled, isFocusable, isFocusOwner, isFocusTraversable, isFontSet, isForegroundSet, isLightweight, isMaximumSizeSet, isMinimumSizeSet, isPreferredSizeSet, isShowing, isValid, isVisible, keyDown, keyUp, list, list, list, location, lostFocus, mouseDown, mouseDrag, mouseEnter, mouseExit, mouseMove, mouseUp, move, nextFocus, paintAll, postEvent, prepareImage, prepareImage, remove, removeComponentListener, removeFocusListener, removeHierarchyBoundsListener, removeHierarchyListener, removeInputMethodListener, removeKeyListener, removeMouseListener, removeMouseMotionListener, removeMouseWheelListener, removePropertyChangeListener, removePropertyChangeListener, repaint, repaint, repaint, resize, resize, setBounds, setBounds, setComponentOrientation, setCursor, setDropTarget, setFocusable, setFocusTraversalKeysEnabled, setIgnoreRepaint, setLocale, setLocation, setLocation, setName, setSize, setSize, show, show, size, transferFocus, transferFocusUpCycle`

**Methods inherited from class java.lang.Object**

`equals, getClass, hashCode, notify, notifyAll, wait, wait, wait`

**Field Detail**

### DEFAULT\_WIDTH

```
public static final int DEFAULT_WIDTH
```

:   the default for width

    **See Also:**: Constant Field Values

---


### DEFAULT\_HEIGHT

```
public static final int DEFAULT_HEIGHT
```

:   the default for height

    **See Also:**: Constant Field Values

---


### DEFAULT\_LEFT

```
public static final int DEFAULT_LEFT
```

:   the default for left

    **See Also:**: Constant Field Values

---


### DEFAULT\_TOP

```
public static final int DEFAULT_TOP
```

:   the default for top

    **See Also:**: Constant Field Values

---


### WIDTH

```
public static final int WIDTH
```

:   default width

    **See Also:**: Constant Field Values

---


### HEIGHT

```
public static final int HEIGHT
```

:   default height

    **See Also:**: Constant Field Values


**Constructor Detail**

### ArffViewerMainPanel

```
public ArffViewerMainPanel(java.awt.Container parentFrame)
```

:   initializes the object

    **Parameters:**: `parentFrame` - the parent frame (JFrame or JInternalFrame)


**Method Detail**

### getParentFrame

```
public javax.swing.JFrame getParentFrame()
```

:   returns the parent frame, if it's a JFrame, otherwise null

    :   **Returns:**: the parent frame

---


### getParentInternalFrame

```
public javax.swing.JInternalFrame getParentInternalFrame()
```

:   returns the parent frame, if it's a JInternalFrame, otherwise null

    :   **Returns:**: the parent frame

---


### setParent

```
public void setParent(java.awt.Container value)
```

:   sets the new parent frame

    :   **Parameters:**: `value` - the parent frame

---


### getMenu

```
public javax.swing.JMenuBar getMenu()
```

:   returns the menu bar to be added in a frame

    :   **Returns:**: the menu bar

---


### getTabbedPane

```
public javax.swing.JTabbedPane getTabbedPane()
```

:   returns the tabbedpane instance

    :   **Returns:**: the tabbed pane

---


### setConfirmExit

```
public void setConfirmExit(boolean confirm)
```

:   whether to present a MessageBox on Exit or not

    :   **Parameters:**: `confirm` - whether a MessageBox pops up or not to confirm exit

---


### getConfirmExit

```
public boolean getConfirmExit()
```

:   returns the setting of whether to display a confirm messagebox or not
    on exit

    :   **Returns:**: whether a messagebox is displayed or not

---


### setExitOnClose

```
public void setExitOnClose(boolean value)
```

:   whether to do a System.exit(0) on close

    :   **Parameters:**: `value` - enables/disables a System.exit(0) on close

---


### getExitOnClose

```
public boolean getExitOnClose()
```

:   returns TRUE if a System.exit(0) is done on a close

    :   **Returns:**: true if a System.exit(0) is done on close

---


### refresh

```
public void refresh()
```

:   validates and repaints the frame

---


### getFrameTitle

```
public java.lang.String getFrameTitle()
```

:   returns the title (incl. filename) for the frame

    :   **Returns:**: the frame title

---


### updateFrameTitle

```
public void updateFrameTitle()
```

:   sets the title of the parent frame, if one was provided

---


### getPanelCount

```
public int getPanelCount()
```

:   returns the number of panels currently open

    :   **Returns:**: the number of open panels

---


### getPanel

```
public ArffPanel getPanel(int index)
```

:   returns the specified panel, `null` if index is out of bounds

    :   **Parameters:**: `index` - the index of the panel **Returns:**: the panel

---


### getCurrentIndex

```
public int getCurrentIndex()
```

:   returns the currently selected tab index

    :   **Returns:**: the index of the currently selected tab

---


### getCurrentPanel

```
public ArffPanel getCurrentPanel()
```

:   returns the currently selected panel

    :   **Returns:**: the currently selected panel

---


### isPanelSelected

```
public boolean isPanelSelected()
```

:   checks whether a panel is currently selected

    :   **Returns:**: true if a panel is currently selected

---


### getFilename

```
public java.lang.String getFilename(int index)
```

:   returns the filename of the specified panel

    :   **Parameters:**: `index` - the index of the panel **Returns:**: the filename for the panel

---


### getCurrentFilename

```
public java.lang.String getCurrentFilename()
```

:   returns the filename of the current tab

    :   **Returns:**: the current filename

---


### setFilename

```
public void setFilename(int index,
                        java.lang.String filename)
```

:   sets the filename of the specified panel

    :   **Parameters:**: `index` - the index of the panel: `filename` - the new filename

---


### setCurrentFilename

```
public void setCurrentFilename(java.lang.String filename)
```

:   sets the filename of the current tab

    :   **Parameters:**: `filename` - the new filename

---


### loadFile

```
public void loadFile(java.lang.String filename)
```

:   loads the specified file

    :   **Parameters:**: `filename` - the file to load

---


### loadFile

```
public void loadFile()
```

:   loads the specified file into the table

---


### saveFile

```
public void saveFile()
```

:   saves the current data into a file

---


### saveFileAs

```
public void saveFileAs()
```

:   saves the current data into a new file

---


### closeFile

```
public void closeFile()
```

:   closes the current tab

---


### closeFile

```
public void closeFile(boolean showCancel)
```

:   closes the current tab

    :   **Parameters:**: `showCancel` - whether to show an additional CANCEL button in the "Want to save changes"-dialog **See Also:**: `saveChanges(boolean)`

---


### closeAllFiles

```
public void closeAllFiles()
```

:   closes all open files

---


### showProperties

```
public void showProperties()
```

:   displays some properties of the instances

---


### close

```
public void close()
```

:   closes the window, i.e., if the parent is not null and implements
    the WindowListener interface it calls the windowClosing method

---


### undo

```
public void undo()
```

:   undoes the last action

---


### copyContent

```
public void copyContent()
```

:   copies the content of the selection to the clipboard

---


### search

```
public void search()
```

:   searches for a string in the cells

---


### clearSearch

```
public void clearSearch()
```

:   clears the search, i.e. resets the found cells

---


### renameAttribute

```
public void renameAttribute()
```

:   renames the current selected Attribute

---


### attributeAsClass

```
public void attributeAsClass()
```

:   sets the current selected Attribute as class attribute, i.e. it moves it
    to the end of the attributes

---


### deleteAttribute

```
public void deleteAttribute(boolean multiple)
```

:   deletes the current selected Attribute or several chosen ones

    :   **Parameters:**: `multiple` - whether to delete myultiple attributes

---


### deleteInstance

```
public void deleteInstance(boolean multiple)
```

:   deletes the current selected Instance or several chosen ones

    :   **Parameters:**: `multiple` - whether to delete multiple instances

---


### sortInstances

```
public void sortInstances()
```

:   sorts the current selected attribute

---


### showAttributes

```
public java.lang.String showAttributes()
```

:   displays all the attributes, returns the selected item or NULL if canceled

    :   **Returns:**: the name of the selected attribute

---


### showValues

```
public void showValues()
```

:   displays all the distinct values for an attribute

---


### setOptimalColWidths

```
public void setOptimalColWidths()
```

:   sets the optimal column width for all columns

---


### actionPerformed

```
public void actionPerformed(java.awt.event.ActionEvent e)
```

:   invoked when an action occurs

    :   **Specified by:**: `actionPerformed` in interface `java.awt.event.ActionListener`
    :   **Parameters:**: `e` - the action event

---


### stateChanged

```
public void stateChanged(javax.swing.event.ChangeEvent e)
```

:   Invoked when the target of the listener has changed its state.

    :   **Specified by:**: `stateChanged` in interface `javax.swing.event.ChangeListener`
    :   **Parameters:**: `e` - the change event

---


### toString

```
public java.lang.String toString()
```

:   returns only the classname

    :   **Overrides:**: `toString` in class `java.awt.Component`
    :   **Returns:**: the classname


---


|  |  |  |
| --- | --- | --- |
| **Overview** \| **Package** \| **Class** \| **Tree** \| **Deprecated** \| **Index** \| **Help** \| **Weka's home** | |  |
| **PREV CLASS**   **NEXT CLASS** | **FRAMES**    **NO FRAMES**     **All Classes** |  |
| SUMMARY: NESTED \| FIELD \| CONSTR \| METHOD | DETAIL: FIELD \| CONSTR \| METHOD |  |


---
